# Mechanics-guided parametric modeling of intranasal spray devices and formulations for targeted drug delivery to the nasopharynx

**DOI:** 10.3389/fddev.2025.1721960

**Published:** 2025-12-12

**Authors:** Md Tariqul Hossain, Abir Malakar, Mohammad Yeasin, William O’Connell, Mohammad Mehedi Hasan Akash, Azadeh A. T. Borojeni, Devranjan Samanta, Gerallt Williams, Joshua Reineke, Gonçalo Farias, Sunghwan Jung, Julie Suman, Saikat Basu

**Affiliations:** 1 Department of Mechanical Engineering, South Dakota State University, Brookings, SD, United States; 2 Department of Mechanical Engineering, Florida State University FAMU-FSU College of Engineering, Tallahassee, FL, United States; 3 Department of Mechanical Engineering, Indian Institute of Technology Ropar, Rupnagar, Punjab, India; 4 Aptar Pharma, Le Vaudreuil, France; 5 Department of Pharmaceutical Sciences, South Dakota State University, Brookings, SD, United States; 6 Department of Biological and Environmental Engineering, Cornell University, Ithaca, NY, United States; 7 Aptar Pharma, Congers, NY, United States; 8 Suman Pharma Solutions, LLC, Columbia, MD, United States

**Keywords:** nasal drug delivery, respiratory transport, intranasal sprays, computational fluid dynamics, large eddy simulation, spray plume angle, formulation density, sprayed particle size

## Abstract

**Introduction:**

Improving the efficacy of nasal sprays by enhancing targeted drug delivery to intra-airway tissue sites prone to infection onset is hypothesized to be achievable through an optimization of key device and formulation parameters, such as the sprayed droplet sizes, the spray cone angle, and the formulation density. This study focuses on the nasopharynx, a primary locus of early viral entry, as the optimal target for intranasal drug delivery.

**Methods:**

Two full-scale three-dimensional anatomical upper airway geometries reconstructed from high-resolution computed tomography scans were used to numerically evaluate a cone injection approach, with inert particles mimicking the motion of sprayed droplets within an underlying inhaled airflow field of 15 L/min, commensurate with relaxed breathing conditions. Therein we have considered monodisperse sprayed particles sized between 10–50 μ m, six material densities ranging from 1.0–1.5 g/mL for the constituent formulation, and twelve plume angles spanning 15 ${\;}^{{}^{\circ}}$ – 70 ${\;}^{{}^{\circ}}$ subtended by the spray jet at the nozzle position. Large Eddy Simulation-based modeling of the inhaled airflow physics within the anatomical domains was coupled with a Lagrangian particle-tracking framework to derive the drug deposition trend at the nasopharynx.

**Results:**

The resulting three-dimensional deposition contour map, obtained by interpolating the outcomes for the discrete test parameters, revealed that the mean nasopharyngeal deposition rate peaked for particle sizes d∈  [25, 45] μ m and plume angles θ≲ 30 ${\;}^{{}^{\circ}}$ , with the deposition rates averaged over the test airway geometries and formulation densities. That mean deposition rate at the nasopharynx was approximately 11.4% within the specified {d,θ} parametric bounds. In addition, the formulation density of 1.0 g/mL yielded the highest mean deposition rate, over the comprehensive tested range of sprayed particle sizes and plume angles. A subset of the simulated nasopharyngeal deposition trends was experimentally validated through representative physical spray tests conducted in a 3D-printed replica of one of the test geometries.

**Discussion:**

The overall findings, while implicitly tied to the two test subjects (i.e., for spray administration through four representative nasal pathways), do collectively demonstrate that rational optimization of the intranasal sprays for targeted nasopharyngeal deposition is attainable with actionable design modifications on the sprayed droplet sizes and device plume angles.

## 1 Introduction

Respiratory viral infections, including influenza, COVID-19, and the common cold, continue to pose major global public health challenges (Volpe et al., 2023). Effective treatment during the initial phase of infection and, in general, prevention are crucial to reducing the impact of these diseases. Intranasal drug delivery systems, especially intranasal sprays (Pires et al., 2009; Popper et al., 2023; Wu et al., 2025), have emerged as a promising method for delivering targeted therapeutic agents, vaccines, and antiviral medications directly to the infected tissue sites along the airway (Afkhami et al., 2022; Mi et al., 2024; Jin et al., 2024; Banella et al., 2025).

**FIGURE 1:**
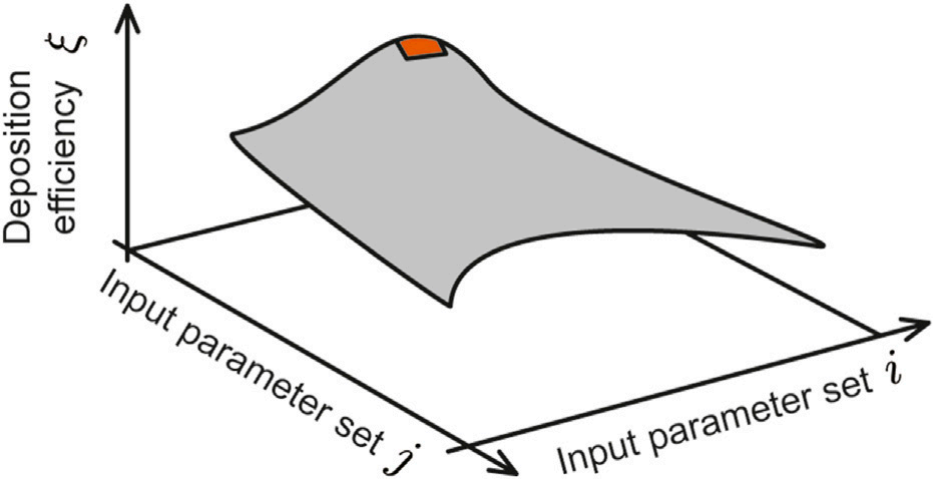
Envisioned 3D contour map: The vertical axis represents the deposition efficiency (ξ) at the target tissue site (in this study, the nasopharynx) and the two horizontal axes represent two controllable input delivery parameters (e.g., spray plume angle of the delivery device, particle sizes of drug solution, nozzle exit speed of the delivery device, viscosity of the solution, delivery axis orientation, drug solution density etc.). For this study, the two chosen input variable axes are sprayed particle size range ( d ; 10–50 μ m) and a range of plume angles for a typical spray-based delivery device ( θ ; 15°–70°). Considering 6 different drug solution densities ( ρ ; 1.0–1.5 g/mL), 24 different contour plots were generated and analyzed for each test airway (number of airways is 4; 2 for each subject); see Figure 6 for a representative subset of the plots. By averaging the data across geometries and test formulation densities (see Figure 7 and specifically Figure 8), this study identifies the equivalent of the red region (illustrated on the cartoon parametric map above) that captures the suitable parametric conditions for maximal target site deposition efficiency.

The nasopharynx—the upper part of the pharynx located at the back of the nose—serves as a critical hotspot for initial respiratory infections via inhaled transmission (Matheson and Lehner, 2020; Hou et al., 2020; Basu, 2021), largely owing to the presence of specific surface receptors that pathogens can exploit for cell invasion, combined with a relatively sparse local mucociliary substrate (Lee et al., 2019). Also note that the nasopharyngeal region contains nasal-associated lymphoid tissue (NALT) (Brandtzaeg, 2011; Laube et al., 2024), which offers a direct connection to the immune system. To enhance the therapeutic efficacy against certain pathogens, such as the SARS and influenza viruses, it could be therefore construed essential to improve the targeted delivery of drugs (Kashyap and Shukla, 2019) at the nasopharynx. With that perspective, this study explores the use of intranasal sprays as a method for targeted drug administration to the nasopharynx and models the transport of sprayed drug particulates during relaxed inhalation (at 15 L/min), through experimentally validated computational simulations of the relevant respiratory flow physics inside two anatomical domains built from medical imaging. We derive the nasopharyngeal deposition efficiency ( ξ , in %) across a broad range of formulation and device parameters, namely the material density of the sprayed formulation ( ρ , in g/mL), the aerodynamic diameters of the sprayed particles ( d , in μ m), and the plume angle of the conical spray discharge ( θ , in degrees) subtended at the nozzle location by the spray jet. The main takeaways from our study will address the following question: *which specific combination(s) of* d *,* θ *, and* ρ *will maximize intra-nasally sprayed drug deposition at the nasopharynx?* To that end, we have derived a three-dimensional parametric diagram (conceptualized in Figure 1) whose topological shape can promptly help identify the parametric conditions for maximal spray performance for targeted delivery at a specific tissue site, such as the nasopharynx.

Maximizing local deposition at infection-prone regions is understandably crucial for improving pharmaceutical effectiveness (Foo et al., 2007; Perkins et al., 2018; Basu et al., 2020; Tong et al., 2016). Traditional methods for optimizing nasal spray formulations and delivery devices often involve trial and error, which can be both time-consuming and costly. Using full-scale three dimensional computational fluid dynamics (CFD) modeling (Feng et al., 2021; Hayati et al., 2023; Hosseini et al., 2020; Dey S. et al., 2025; Islam and Rahman, 2025; Niegodajew, 2025; Basu, 2021; Kleinstreuer et al., 2008), it is however possible to reliably simulate how drug particles behave as they move through the tortuous nasal passages (Basu et al., 2018; Kleven et al., 2012; Farnoud et al., 2020). These models can predict tissue-specific regional deposition patterns based on factors such as sprayed droplet sizes, spray plume angles, formulation properties, and airflow conditions—thereby offering valuable insights into how to finetune the design of intranasal sprays for better targeting efficacy (Inthavong et al., 2008). Herein, we use the same approach to guide the optimization of current formulations along with laying the groundwork for developing CFD-informed augmented intranasal delivery systems. The intra-airway dynamics of the sprayed droplets was modeled by assuming them as inert discrete phase particles bearing appropriate physical properties (in terms of spherical shapes/sizes, material density). For clarity, as we move further into the exposition—the reader should note that the terminologies “droplets” and “particles” have been used interchangeably in this paper.

### 1.1 As a sequel to our last nasal spray study at this journal

Systematically pinning down the droplet transport features and the resulting deposition patterns within realistic nasal cavities is crucial toward designing new-generation sprays that can effectively target the disease-prone tissue regions along the airway. The findings reported here build on our previous publication in this journal (Akash et al., 2023). While the prior study had primarily focused on refining the spray axis orientation and nozzle position within the anterior respiratory airspace for improved targeted drug delivery at the nasopharynx and had used a constrained range of particle sizes, the current work employs simulations validated through realistic physical experiments, comprehensively tests a broad range of parametric conditions for {d,θ,ρ} , and invokes the same spray placement protocol (as in Akash et al. (2023)) to provide significant updates on improving device and formulation design. It figures out the ideal parametric range of sprayed droplet sizes, spray plume angles, and sprayed formulation’s material densities that could potentially maximize targeted nasopharyngeal deposition. In essence, the primary motivation for this new study has been to derive a parametric contour map (conceptualized in Figure 1), that can offer a direct mechanistic feedback and actionable design recommendations on the ideal spray and formulation parameters that would enhance drug droplet deposition at the nasopharynx (it being the target tissue site) by intranasal spray delivery systems.

Preliminary results from this work have been presented at the Annual Meetings of the American Physical Society’s Division of Fluid Dynamics (Malakar et al., 2023; Hossain et al., 2025; O’Connell et al., 2025). As a caveat though, the reader should note that the findings derived in the subsequent sections on the ‘optimal’ (or, ideal) spray and formulation designs are based on data obtained by simulating respiratory transport in only two adult subjects, therefore comprising results from spray administration through four different nasal pathways.

## 2 Materials and methods

### 2.1 Anatomical domain reconstruction

**FIGURE 2:**
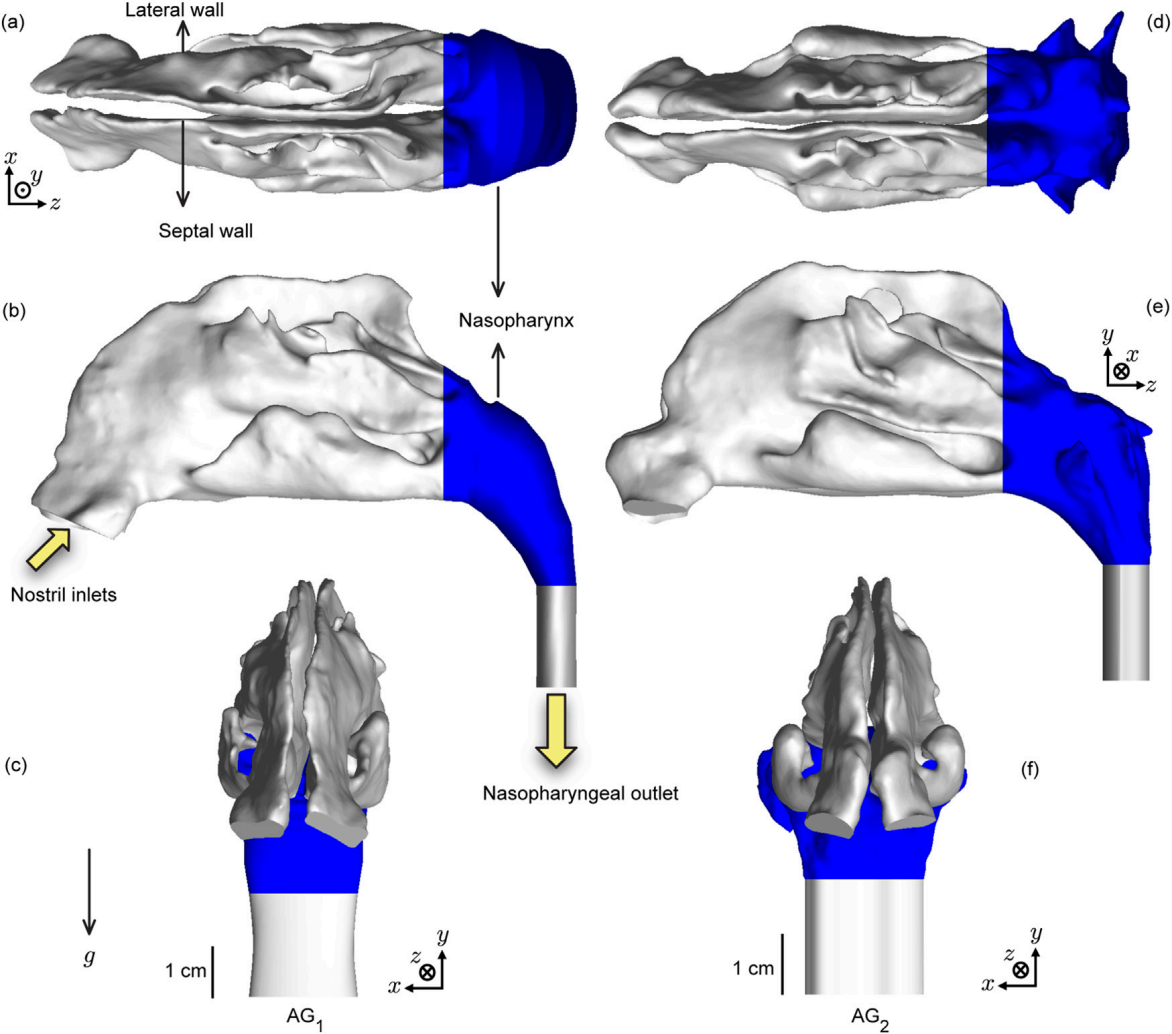
Test upper airway geometries: Panels **(a–c)** respectively show the axial, sagittal, and coronal views of the computed tomography (CT)-based anatomical reconstruction of ${\text{AG}}_{1}$ (anatomical geometry 1). Similarly, panels **(d–f)** respectively show the axial, sagittal and coronal views of the CT-based reconstruction of ${\text{AG}}_{2}$ (anatomical geometry 2). The nasopharynx is colored blue in the visuals (Borojeni et al., 2017). The solid yellow arrows indicate the airflow inlet and outlet regions for the inhalation simulations, with their directions aligning with the direction of mean flux. g implies the gravity direction in the simulations. Additionally, the visuals in **(c,f)** show the geometry-specific length scales. **(a–c)** have the same scale and **(d–f)** have the same scale.

The anatomical upper airway geometries (see Figure 2), used in this study, were rebuilt from existing, de-identified, medical-grade computed tomography (CT) imaging data collected from two adult test subjects with disease-free airways. Therein, the coronal depth increments in between the CT slices were ≈ 0.4 mm. We have named the test domains as anatomical geometry 1 (or, ${\text{AG}}_{1}$ ) and anatomical geometry 2 (or, ${\text{AG}}_{2}$ ). The computational retrospective use of the existing, anonymized scans was approved with exempt status by the Institutional Review Board at South Dakota State University. For anatomical precision, the nasal airspace segmentation was carefully hand-edited after applying the expected radio-density delineation of −1024 to −300 Hounsfield units (Basu et al., 2018). For this step, the high-resolution DICOM (Digital Imaging and Communications in Medicine) scans for each subject were imported into the image-processing program Mimics Research 18.0 (Materialise, Plymouth, MI).

Subsequently, we imported the reconstructed geometries to ICEM CFD 2024 R1 (ANSYS Inc., Canonsburg, Pennsylvania) as stereolithography (STL) files. To spatially mesh the anatomical cavities according to established mesh refinement-based protocols (Frank-Ito et al., 2016; Basu et al., 2017), each computational grid included 3 prism layers ( ≈ 0.1 mm thick) along the airway walls (except the nostril inlet planes and the outlet plane) with a height ratio of 1. For the core cavity space, approximately 4.2 million (in ${\text{AG}}_{1}$ ) and 4.4 million (in ${\text{AG}}_{2}$ ) unstructured, tetrahedral elements were generated by implementing the tetra/mixed type mesh with robust (Octree) method. Combined with the prismatic element counts, the total element numbers were 5.3 million in ${\text{AG}}_{1}$ and 5.4 million in ${\text{AG}}_{2}$ .

Spray axis determination and nozzle placement: The spray placement in the digitized airspace domains followed the “line-of-sight” (LoS) protocol established by us previously (Basu et al., 2020; Akash et al., 2023; Treat et al., 2020) for improved drug delivery, whereby the spray axis should (virtually) cut through the target tissue site. Accordingly, after ascertaining the centroid of the nostril plane (through which spray would be administered) in each reconstructed geometry, we identified an arbitrary point generally positioned near the upper edge of the nasopharynx. The direction vector between the nostril centroid and the located point provided a repeatable spray direction, with the spray nozzle placed 5-mm into the airspace from the nostril centroid. Note that the nasopharynx comprises the upper segment of the pharynx at the back of the nose, after the two sides of the anterior nasal airspace merge; see Figure 2.

### 2.2 Numerical simulations

#### 2.2.1 Inhaled airflow and sprayed particle tracking simulations

**FIGURE 3:**
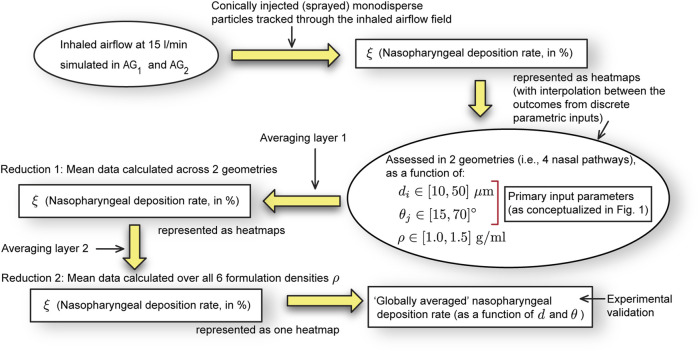
Schematic workflow: This computational study assesses the nasopharyngeal deposition rates (ξ) for discrete combinations of formulation densities (ρ) , plume angles (θ) , and sprayed particle diameters (d) . The deposition heatmaps (included in the results) give interpolated visuals from the discrete assessments. As per the schematics in Figure 1, the input parameter i and input parameter j are d and θ (in no specific order). We generate ξ for a wide range of d and θ , for six specific ρ . Eventually, the “globally averaged” ξ is obtained, by averaging the nasopharyngeal deposition assessments for all ρ and test geometries (with two layers of averaging, as illustrated above).

This study investigated the intra-airway deposition behavior of 3,000 monodisperse particles—each set bearing aerodynamic diameters d∈[10,50]μ m (with increments of 1 μ m). These particles were sprayed into the anatomical domains carrying an underlying airflow field that mimicked resting inhalation rate of 15 L/min (Garcia et al., 2009; Borojeni et al., 2020). The exercise was performed for six formulation densities, ρ∈[1.0,1.5] g/mL (with increments of 0.1 g/mL) and twelve plume angles, θ∈[15°,70°] (with increments of 5 ${\;}^{{}^{\circ}}$ ); see Figures 1, 3 for the conceptualized study design. A cone injection technique was employed to ensure realistic drug delivery and reliable particle deposition measurements.

The inhalation airflow was modeled using the Large Eddy Simulation (LES) scheme that resolved turbulent flow structures, dividing the turbulence (Longest and Vinchurkar, 2007; Doorly et al., 2008) into large- and small-scale motions. Subgrid-scale kinetic energy transport model was invoked to track small fluctuations (Baghernezhad and Abouali, 2010; Farnoud et al., 2020). The computational simulations were performed on ANSYS Fluent 2024 R1, with the implementation of a segregated solver. Therein we used the SIMPLEC pressure-velocity coupling and second-order upwind spatial discretization. The solution convergence was monitored by minimizing the mass continuity residuals to $10^{-2}$ and velocity component residuals to $10^{-6}$ . Additionally, the stabilization of mass flow rate and static pressure at airflow outlets, namely at the nasopharyngeal outlet (see Figure 2), was kept track of. For these pressure gradient-driven airflow solutions, the LES work necessitated a computing time of 1.5–2 days, to replicate inhalation flow over a span of 0.35 s (adequate for sprayed particle transport to cover the nostril-to-nasopharynx pathway), using a time-step of 0.0002 s (Ghahramani et al., 2017). To accurately mimic warmed-up air inside the respiratory route, its dynamic viscosity was set at $1.825\times 10^{-5}$ kg/m.s and the density was assumed as 1.204 kg/m^3^.

**FIGURE 4:**
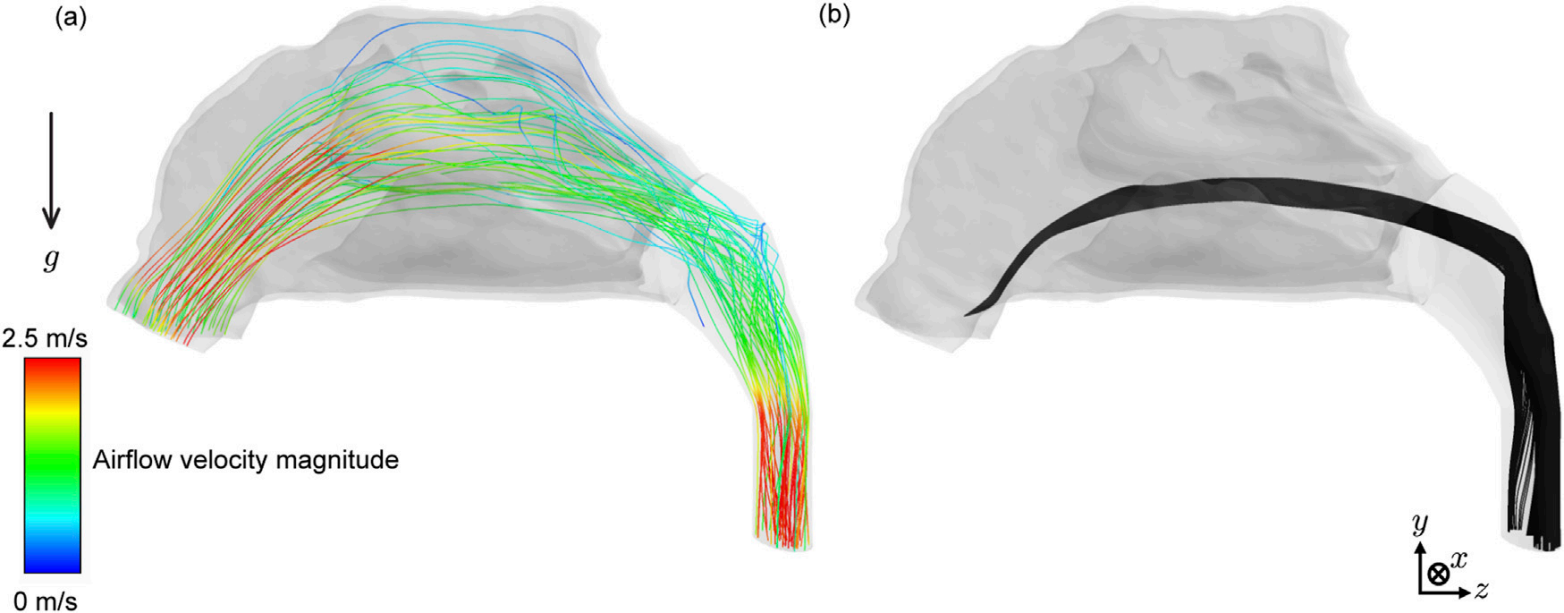
Representative flow field and sprayed particle trajectories: **(a)** Sample airflow velocity streamlines within ${\text{AG}}_{1}$ mimicking 15 L/min inhalation. These representative streamlines initiate from 25 random points on each nostril (i.e., a total of 50 streamlines are shown above). **(b)** Sample spatial trajectories of intra-nasally sprayed particles, with ρ= 1.0 g/mL, θ= 15°, and d= 18 μ m.

The tracking of intranasal spray dynamics against the surrounding inhaled airflow was accomplished using Lagrangian-based inert discrete phase simulations (e.g., see Figure 4) with Runge-Kutta solver. The motion of the sprayed particles was assumed to be one-way coupled with the surrounding flow (Inthavong et al., 2008; Feng et al., 2017; Zhao et al., 2021), meaning that the particles’ trajectories were influenced by the flow features, but they did not, in turn, affect the airflow field around them. The simulations integrated the particle transport equation that accounted for various forces acting on small particulates, such as the ambient inhaled airflow drag, gravity, and other appreciable body forces (namely the Saffman lift force relevant for small particles). While deriving the particle deposition data, we implemented a no-slip trap boundary condition on the walls of the cavity, enabling the assessment of localized droplet clustering over intranasal tissues. For each formulation density, the sprayed droplets (also often referred to as “particles” in this study) were introduced into the airspace as a solid-cone injection starting from the nozzle point. The initial velocity of the droplets was realistically set at 10 m/s (Liu et al., 2011) and a total non-zero mass flow rate of $1\times 10^{-20}$ kg/s was given as the initial condition of the streams in the spray cone.

The following expands on the boundary conditions during particle tracking: (i) The airway-tissue interface, which represented the walls enclosing the digitized nasal airspace, had a zero tangential velocity condition (commonly known as the “no-slip” condition); additionally, the walls were enforced with the “trap” discrete phase boundary condition, enabling the particle tracks to cease once they enter the elements adjoining the walls. (ii) For the nostril planes, a “reflect” discrete phase boundary condition was used to simulate the effect of inhalation on the particle trajectories if they were on the verge of falling out of the anterior nasal domain. (iii) The airflow outlet plane, designated as the pressure-outlet zone, had an “escape” discrete phase boundary condition, allowing the outgoing particle trajectories to exit the upper respiratory airspace. Considering the area-weighted average of the inlet and outlet pressure variables in the simulations, the mean total pressure gradient driving the 15 L/min airflow in the two test geometries was 5.63 Pa (with a strikingly comparable 5.66 Pa in ${\text{AG}}_{1}$ and 5.59 Pa in ${\text{AG}}_{2}$ ).

For details on the mathematical formalism for the numerical scheme employed in this study, please refer to Basu (2025). The computational approach has also been thoroughly validated in one of our earlier publications (Basu et al., 2020). This validation involved comparing the regional deposition patterns along the inner walls of *in silico* nasal anatomical models with gamma scintigraphy measurements of regional deposition obtained from *in vitro* spray tests conducted in 3D-printed solid transparent replicas with similar reconstructions.

### 2.3 Experimental setup

**FIGURE 5:**
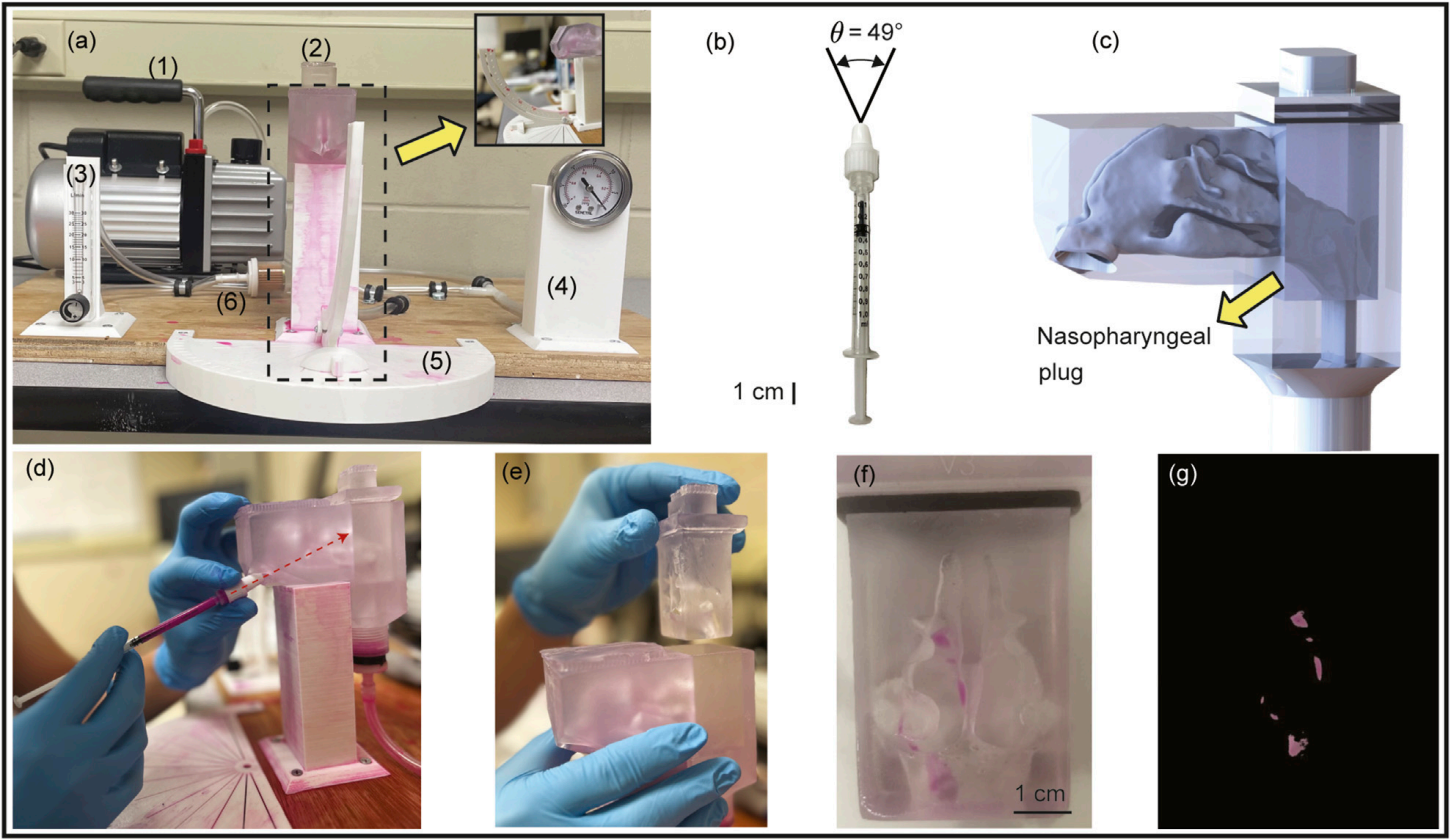
Setup for experimental validation: Panel **(a)** shows the front view of the experimental setup, comprising the following numbered components: (1) a 2.5 CFM vacuum pump, (2) a 3D-printed nasal airway model, (3) a flow rate meter, (4) a pressure gauge, (5) a spray cone angle indicator, and (6) an air filter. Inset shows the side view of the setup. Panel **(b)** demonstrates the LuerVax^TM^ spray device (an Aptar Pharma product) used in the experiments. The spray plume angle is θ=49° . Panel **(c)** presents the 3D CAD visual of the nasal airway cast ready to be printed, with the nasopharyngeal region constructed as a removable plug system for ease of measuring the local deposits. See Figure 2f, for the length scale of the life-sized anatomical reconstruction. Panel **(d)** illustrates the procedure of the experimental spray trials, with the dotted red arrow representing the spray axis cutting through the nasopharynx. Panel **(e)** shows the removable nasopharyngeal plug. Panel **(f)** provides a representative image taken after one of the spray trials, while panel **(g)** shows the corresponding image-processed view of **(f)**.

**FIGURE 6:**
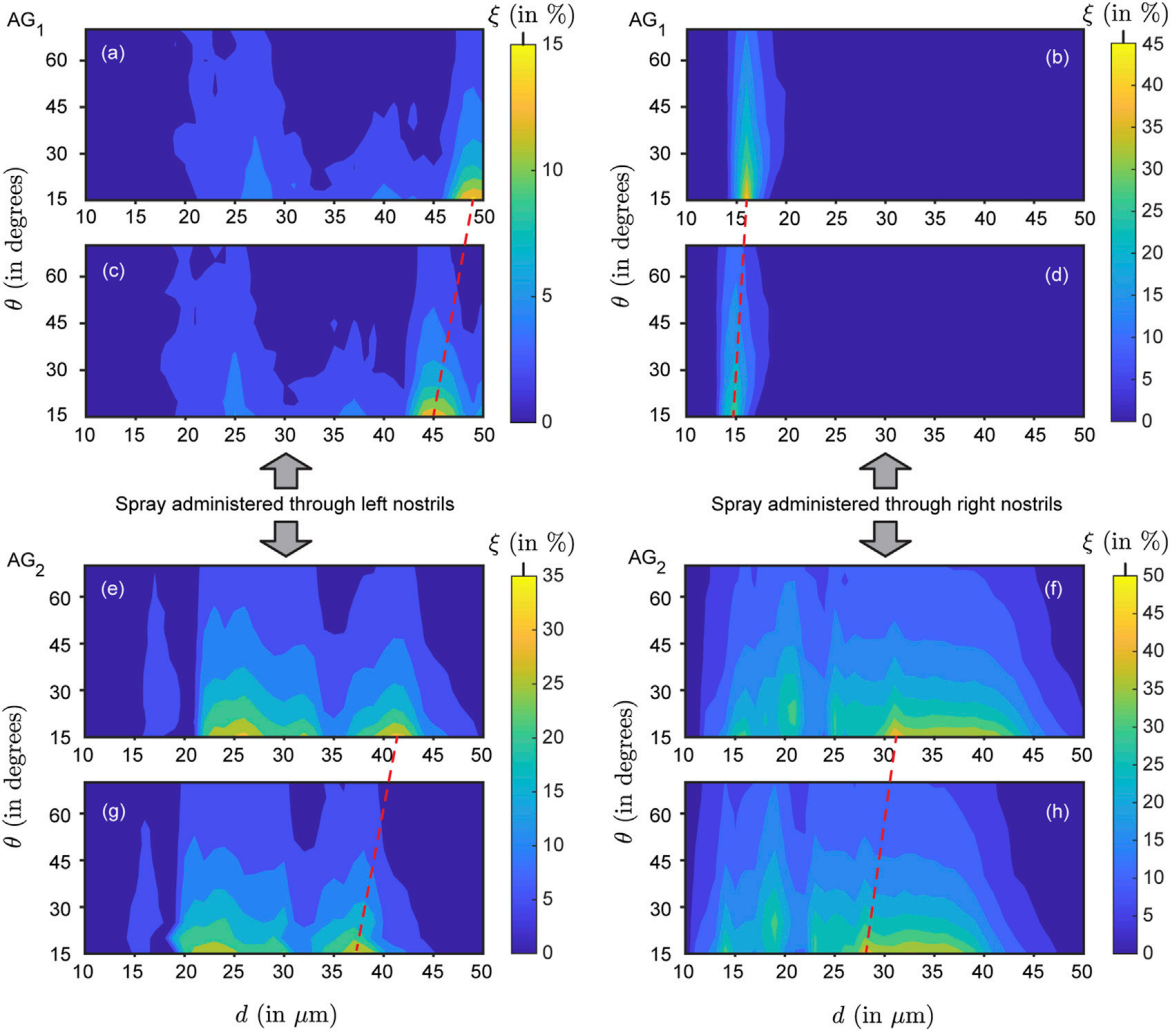
Representative simulated nasopharyngeal deposition trends for ${\text{AG}}_{1}$ (panels **(a–d)**) and ${\text{AG}}_{2}$ (panels **(e–h)**): Contour plots illustrating the nasopharyngeal deposition rate (defined as the fraction of monodisperse particles depositing at the nasopharynx; denoted by ξ ) as a function of the spray plume angles ( θ , recorded along the vertical axis) and particle diameters ( d , along the horizontal axis). Panels **(a,b)** and **(e,f)** are for ρ=1.2 g/mL, while panels **(c,d)** and **(g,h)** are for ρ=1.4 g/mL. With higher inertia thwarting downwind penetration, the optimal parametric region (for maximal ξ ) gradually shifts toward the left side of the contour map with increasing formulation density ρ . The shifts are indicated by the red dashed lines, visibly inclined. Note that the paired panels **(a–c,b–d,e–g,f–h)** are aligned horizontally and share the same vertical spacing (between the paired panels), ensuring consistent inclination angles of the dashed lines across pairs. The left column (of panels) depicts data for spray delivered through the left nostril; the right column shows data for spray through the right nostril. Overall, for all densities (ρ) , the mean ξ in ${\text{AG}}_{1}$ —when sprayed through the right nostril—is lower than that when sprayed through the left nostril. Conversely, in ${\text{AG}}_{2}$ , the mean ξ through the right nostril exceeds that through the left. This exemplifies typical respiratory airspace asymmetries and the uniqueness of each nasal pathway without any specific right/left dependence across subjects. See Supplementary Figures S1, S2 for the comprehensive set of contour maps in ${\text{AG}}_{1}$ and ${\text{AG}}_{2}$ , ∀ ρ .

With ${\text{AG}}_{2}$ demonstrably exhibiting higher nasopharyngeal deposition (per the simulations; see Section 3, Figure 6), a 3D-printed cast of ${\text{AG}}_{2}$ was built for physical experimental tests, with sprays administered through its both nasal openings. The experimental setup (see Figure 5a) included a 2.5 cubic feet per minute (CFM) vacuum pump (Pittsburgh Automotive®, 120VAC/60 Hz/3.2A), a flow rate meter connected to an air filter, a pressure gauge, a stable platform for securing the geometry model, and a spray cone angle indicator. The experiments were conducted using LuerVax^TM^ spray device (see Figures 5b,d) filled with a fluorescent dye solution diluted in distilled water, with the solution having an approximate density of 1.054 g/mL and the spray administered with a measured plume angle of θ= 49 ${\;}^{{}^{\circ}}$ (see Figure 5b; also see Table 1), with hand actuation (see Figure 5d) while targeting the nasopharynx following the described LoS protocol.

**TABLE 1:** Spray parameters: Mean droplet size distribution (DSD) and plume angles (θ) in Aptar Pharma spray products for intranasal vaccination (targeting the nasopharynx). Errors on θ are a measure of the corresponding standard deviations in the measurements (with 5 trials for each product). The DSD was determined via laser diffraction using a Malvern Spraytec^®^ (Malvern Instruments, Worcestershire, United Kingdom). θ was evaluated using a SprayVIEW^®^ (Proveris Scientific; Hudson, MA) measuring system, which is a non-impaction laser sheet-based instrument. For the listed measurements, the atomizer was positioned 5 cm from the laser for plume angle measurements and 4 cm from laser for the DSD measurements (Baxter et al., 2022). Detailed parameters associated with the measurement techniques are available in Laube et al. (2024).

Product	${\text{Dv}}_{10}$ ( μ m)	${\text{Dv}}_{50}$ ( μ m)	${\text{Dv}}_{75}$ ( μ m)	${\text{Dv}}_{90}$ ( μ m)	θ (degrees)
BiVax 200^TM^	19	36	49	64	69 ± 2
LuerVax^TM^	20	43	64	89	49 ± 1

The 3D geometry of ${\text{AG}}_{2}$ was printed in a stereolithography (SLA) 3D printer, Anycubic Photon M3 Max, by employing high-clear resin in an attempt to yield optical transparency. The optical transparency of the resulting resin was critical, as it enabled visualization of post-deposition patterns through fluorescent excitation. SLA printing was chosen over other forms of additive manufacturing (He et al., 2019)—given its ability to yield higher resolution, smooth finish over the whole surface, as well as higher fidelity in replicating complex internal anatomical structures.

In addition, the nasopharyngeal portion (see Figure 2) was fabricated in the form of a removable plug (shown in Figures 5c–f), and instead of re-using the same plug, 20 different plugs were used during 10 spray trials run through each nostril. The spray administration protocol (comprising 10 trials in each nasal opening; the nozzle being ≈ 5-mm into the airspace along the LoS direction) was carefully tuned for a robust quantification of the deposited mass at the nasopharynx. The use of clear resin also minimized optical scattering, therefore further permitting more reliable as well as reproducible quantification of deposition levels. Following each individual test, high-resolution images of the anterior, posterior, and lateral portions of the plug were captured. These images were processed in MATLAB using color masks (e.g., see Figures 5f,g) to generate a percentage of deposition relative to the image. Each trial had the sum of its depositions calculated from MATLAB to quantify dye deposition within the nasopharyngeal plug.

## 3 Results

### 3.1 Numerical simulation results

#### 3.1.1 Variation trend in nasopharyngeal deposition

**FIGURE 7:**
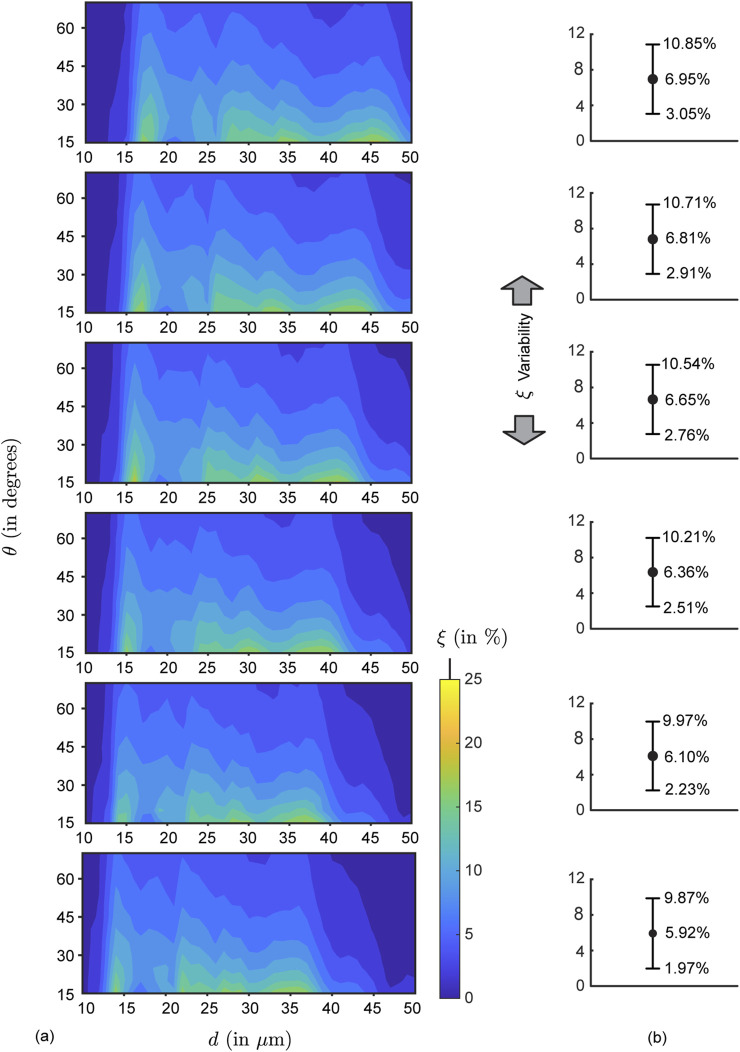
Mean simulated deposition rates for each test formulation density: **(a)** Contour maps for mean nasopharyngeal deposition rate (ξ) for each test formulation density, ρ , which varies from 1.0 g/mL to 1.5 g/mL (with increments of 0.1 g/mL), depicted progressively from top panel to the bottom panel. The averaging considers both nasal sides of ${\text{AG}}_{1}$ and ${\text{AG}}_{2}$ . **(b)** Variability plots showing the mean ξ (marked by the solid black circles, corresponding to the data from the respective left panel), with the error range based on the corresponding standard deviations of the simulated ξ in each parametric case.

In our study, six different formulation densities were used, resulting in a total of 24 individual contour maps illustrating simulated nasopharyngeal deposition rates ( ξ , in %) across the tested airways (see Figure 6 for a representative subset of contour maps). Comparing the panels top-to-bottom, the variations (note the leftward shift) are primarily owing to the particles becoming inertially stronger with increasing formulation density. Also, the topological differences on the contour maps along each row (i.e., for left versus right nostril spray administration) indicate the subtle dependence of the local deposition trend on anatomy-specific geometric features. Next, moving beyond the geometric subjectivity, Figure 7 maps ξ when averaged across all four airspaces, for each test formulation density ( ρ∈[1.0,1.5] g/mL, with increments of 0.1 g/mL on panels therein from top-to-bottom).

To expand further on the physics-guided trends, focusing on each column of panels in Figure 6’s contour maps (i.e., for data from the same airway as ρ is progressively raised), we observe that the peak ξ regions in the plots gradually shifted to the left side because of the inertial effect of the formulation densities (higher the density of the solution, higher would be the particle inertia). As shown in Figure 6, with higher inertia through density increase, particles of similar sizes impact and deposit on the airway walls in the anterior tract, resulting in less penetration to the nasopharynx (located at the posterior part of the upper airspace). This behavior can be explained through the Stokes number $Stk=\rho d^{2}U/18\mu D$ (not considering slip correction), where ρ = particle material density, d = particle diameter, U = characteristic fluid speed, μ = viscosity coefficient of the underlying fluid (air), and D = characteristic cavity diameter. Physically, Stk represents the ratio between local transient inertia and ambient fluid viscosity. Increase in ρ enhances the inertial dominance in particle motion. Everything else (including the diameters) staying same—the higher inertial particles are more likely to settle in the front sections of the nose owing to inertial impaction (coupled with gravitational sedimentation), rather than moving further downwind into the complex nasal passage to reach the nasopharynx. Conversely, particles with Stk≲ 1 are carried more efficiently into the deeper airspace by the fluid streamlines they are embedded in (Aggarwal et al., 1994; Dey R. et al., 2025; Schroeter et al., 2011; Finlay, 2001).

#### 3.1.2 Generic parametric bounds for enhanced ξ

**FIGURE 8:**
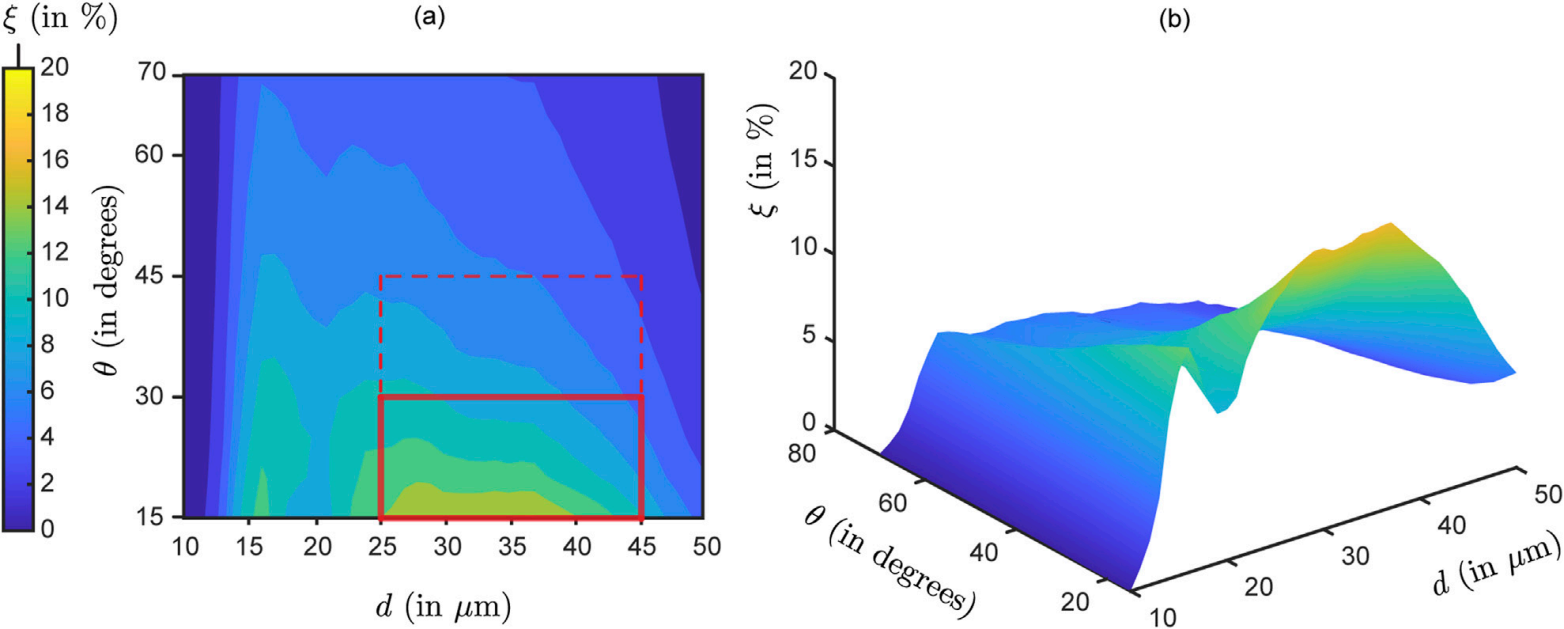
Optimal parametric choices specific to the test domains ${\text{AG}}_{1}$ and ${\text{AG}}_{2}$ : Panels **(a,b)** depict the “globally averaged” contour map for nasopharyngeal deposition rate ( ξ , in %), as the input parameters d and θ are varied. Panel **(b)** is the isometric view of the same data shown in **(a)**. The simulated data here is obtained by averaging across all the test airways and formulation densities. The rectangle bounded by solid red lines in **(a)** demarcate the parametric choices d∈[25,45]μ m and θ≤30° , when ξ visibly peaks. The mean ξ is 11.37% within this region. If we extend the vertical bound to θ≤45° (marked by the dashed red lines), the mean ξ reduces slightly to 9.38%. Compare these to the mean deposition rate for each of the formulation densities throughout the (d,θ) domain, as shown in Figure 7b. The average from the 6 recorded means therein is 6.47%. Note: The reader may find it intriguing to compare the visual in panel **(b)** above to the hypothesized conceptual diagram pitched in Figure 1.

Figures 8a,b show the nasopharyngeal deposition rate ( ξ , in %) when ‘globally’ averaged across all the test airway domains and formulation densities; the flowchart for the averaging algorithm is shown in Figure 3. Within the red rectangle (bounded by solid lines) in Figure 8a where ξ visually peaks, the maximum and minimum ξ are respectively 16.24% and 5.55%. The mean deposition rate within the same parametric bounds with d∈[25,45]μ m and θ≤30° (within the red solid line-bounded rectangle) is 11.37% (averaged from the ξ outcomes for the discrete (d,θ) inputs within the said domain). The latter is 76% higher than the global mean ξ across the entire parametric domain (see Figure 7b). It is worth noting that these particles within the red rectangle are noticeably larger than the typical aerosol dimension of 5 μ m. Particles smaller than 10 μ m would predominantly bypass the nose, penetrating downwind to deposit in the laryngotracheal cavity and the bronchial recesses (Crowder et al., 2002; Chakravarty et al., 2022; Darquenne et al., 2022; Basu, 2025). From a regulatory and manufacturing perspective in context to designing optimized spray formulations for upper airway target sites (such as the nasopharynx), the findings will thus mitigate a potential caveat, as the United States Food and Drug Administration (US FDA) diligently monitors the proportion of droplets measuring less than 10 μ m to ensure safety.

#### 3.1.3 Impact of spray plume angle θ

The geometric features of the spray delivery system are crucial for directing particles to the desired intra-airway locations. The plume angle θ is the solid cone angle subtended at the delivery device nozzle that sprays the pharmaceutical agents (e.g., see Figure 5b). We have investigated 12 different plume angles for both geometries ranging from 15° to 70° (with an increment of 5°). Figures 6–8 show that there is, in general, an inverse relation between the plume angles (θ) and nasopharyngeal deposition rates (ξ) . Higher plume angles result in lower deposition ∀ ρ , in both subjects ( ${\text{AG}}_{1}$ and ${\text{AG}}_{2}$ ). This is expected as a narrower, more pin-pointed spray profile can understandably deliver drugs more efficiently downwind, given the spray axis is directed aptly at the region of interest. Enforcing a threshold of around 5% deposition, it is observed that θ≤60° can yield substantial results for particle sizes falling within the 20–45 μ m range. A more effective choice is θ≤30° , which (as shown in Figure 8a) leads to a mean ξ that exceeds 11%, for d∈[25,45]μ m.

### 3.2 Statistical analysis with uncertainty quantification for the parametric choices deemed suitable for enhanced nasopharyngeal deposition

Table 2 summarizes nasopharyngeal deposition efficiency ( ξ , in %) averaged across all simulated airspace pathways and tested particle densities for five particle diameters ( d∈[25,45] μ m with increments of 5 μ m, identified as the ideal particle size range for enhanced nasopharyngeal deposition; see Figure 8) at two plume angles θ = 30 ${\;}^{{}^{\circ}}$ and 35 ${\;}^{{}^{\circ}}$ (with 30 ${\;}^{{}^{\circ}}$ being projected as the upper threshold for maximal targeted deposition, per Figure 8). For each selected d , Table 2 reports the mean ξ , its standard deviation σ (reflecting variability across pathways and densities), and Cohen’s d as a standardized effect-size comparing θ = 30 ${\;}^{{}^{\circ}}$ versus θ = 35 ${\;}^{{}^{\circ}}$ ; see Table 2’s caption detailing the related calculation. Herein, the Cohen’s d values range from 0.33 to 1.78, with a mean of ≈ 1.03, indicating medium-to-large effects overall and especially large effects for smaller particles ( d∈[25,35] μ m). Because the study uses a small cohort of simulation instances per condition, classical null-hypothesis tests (e.g., t-tests) will have limited power and p -values alone would be unstable; therefore we have employed uncertainty quantification through effect sizes. The large Cohen’s d for particle sizes d∈[25,35] μ m suggests a practically meaningful decrease in ξ when θ increases from 30 ${\;}^{{}^{\circ}}$ to 35 ${\;}^{{}^{\circ}}$ , despite limited sample size, whereas the smaller Cohen’s d values at d∈[40,45] μ m indicate weaker effects. Overall, this analysis helps establish the selection of θ≲30° as the optimal plume angle domain for enhanced nasopharyngeal delivery, with the implicit caveat that this result is based on data from two anatomical geometries (and hence, accounting for spray administration through four representative nasal pathways).

**TABLE 2:** Statistical test: Measure of effect size analysis for the evolving trend in nasopharyngeal deposition efficiency ξ (in %) as the plume angle θ changes from 30 ° to 35 ° . The mean ξ is calculated by averaging the simulated nasopharyngeal deposition data from all the airspace pathways (i.e., ${\text{AG}}_{1}$ left, ${\text{AG}}_{1}$ right, ${\text{AG}}_{2}$ left, ${\text{AG}}_{2}$ right) and all tested values of ρ , keeping the d (selected based on the optimal range reported in Figure 8a) and θ fixed as per values in the tabulated cells herein. σ denotes standard deviation of the discrete ξ data in each test set. The Cohen’s d is calculated as = $\left(M_{30}-M_{35}\right)/\sigma_{\text{pooled}}$ , where $\sigma_{\text{pooled}}=\sqrt{\left({\sigma_{30}}^{2}+{\sigma_{35}}^{2}\right)/2}$ (see Cohen, 2013). $M_{30}$ , $M_{35}$ , $\sigma_{30}$ , and $\sigma_{35}$ are explained in the tabulated cells.

d (in μ m)	θ=30°		θ=35°		Cohen’s d
	$M_{30}$ = mean ξ	$\sigma_{30}=\sigma \left(\xi \right)$	$M_{35}$ = mean ξ	$\sigma_{35}=\sigma \left(\xi \right)$	
25	10.476	1.010	9.274	0.982	1.207
30	9.881	0.968	8.633	1.013	1.260
35	8.989	0.702	7.829	0.598	1.779
40	7.626	2.111	6.565	1.747	0.548
45	5.554	2.835	4.688	2.388	0.330
Mean Cohen’s d =					1.025

### 3.3 Representative experimental validation

**FIGURE 9:**
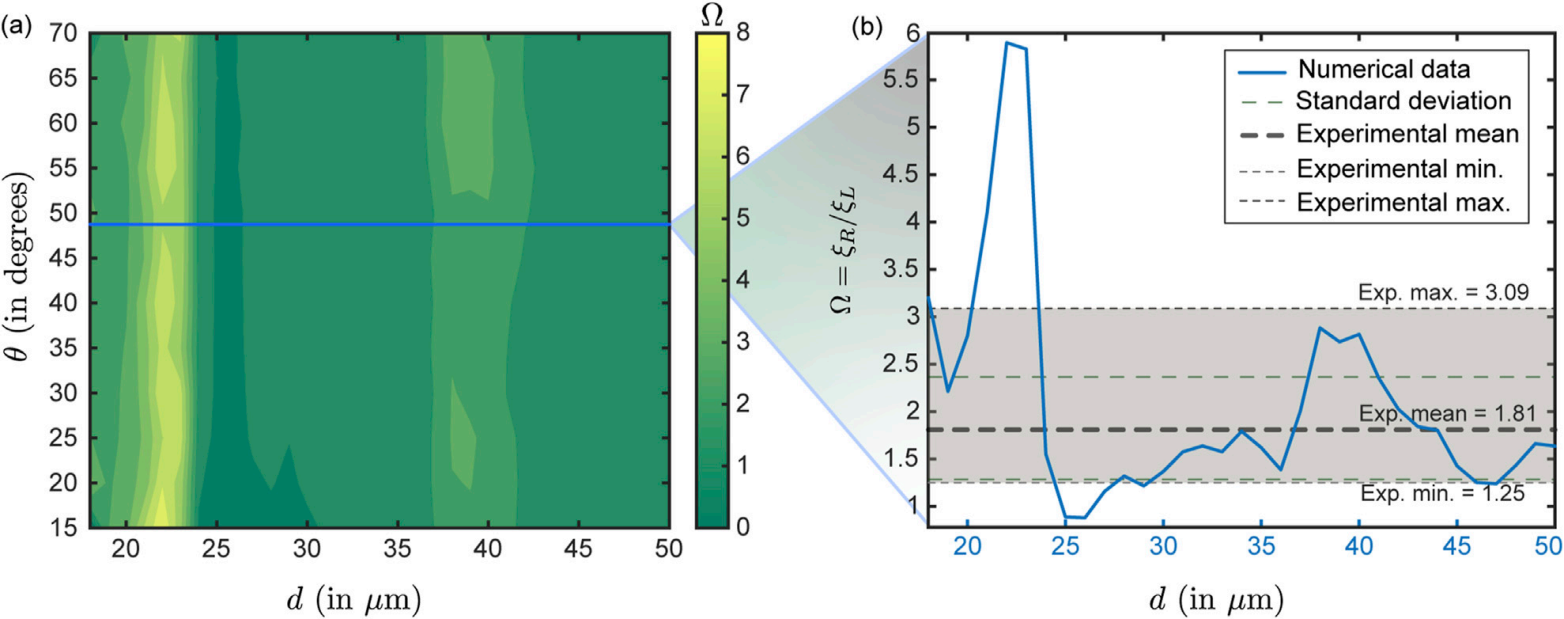
Comparison between numerical and experimental test cases: Panel **(a)** presents the contour plot obtained from numerical simulations showing the ratio of right $\left(\xi_{R}\right)$ – to–left $\left(\xi_{L}\right)$ nostril deposition efficiencies (symbolically represented as Ω ) at nasopharynx for particles with a material density of 1.0 g/mL, corresponding to the experimental condition (with the sprayed formulation particulates comprising water embedded with fluorescent markers and bearing a material density of ≈ 1.05 g/mL). The blue horizontal line (in panel **(a)**) marks the simulation data for the plume angle of 49 ° , which is the measured θ for the LuerVax^TM^ nozzle (an Aptar Pharma product) used in the experiments. Using the blue line, panel **(b)** illustrates the simulated variation of the right-to-left nostril deposition ratio (at nasopharynx) for the test particle sizes for this specific plume angle. The shaded light-gray region denotes the experimentally measured right-to-left deposition ratio range. The thick gray dashed line indicates the experimental mean which is 1.81, the upper and lower light dotted lines respectively represent the maximum (3.09) and minimum values (1.25) from the experimental trials, and the light dashed green lines denote the standard deviation on the experimental Ω . The reader should note that the gray horizontal band in **(b)** represents data from physical experiments conducted with a realistic droplet size distribution in each actuation. It does not correspond directly to the d values (shown in blue) along the horizontal axis. In contrast, the simulation-derived Ω (blue plot) are functions of d , since the simulations assume monodisperse droplets. It is reassuring that the simulation results and experimental data agree when d≳25 μ m, as the experimental spray shots are intentionally composed of particles dominated by that size range (e.g., refer to Table 1).

Physical experiments comprising nasal spray administration (mimicking the computational spray delivery protocol, with LuerVax^TM^) were performed 10 times per nostril within the 3D printed cast of ${\text{AG}}_{2}$ . Each test consisted of an airflow rate of 15 L/min passing through the anatomical cast (mimicking inhalation; e.g., see Figure 5) and ten pumps of fluorescent solution from the spray bottle inserted into the printed model (see Section 2.3 for details on the sprayed solution). For each trial, the deposition rates for the right nostril are found higher than those for the left nostril; this trend is in agreement with the numerical results for ${\text{AG}}_{2}$ (see Figures 6e–h, 9a). Each trial then compared the respective nasopharyngeal depositions for spray administration through the two nostrils to generate a ratio. During the ten trials, the ratio (represented as Ω ) of the experimental ξ when sprayed through the right nostril to the ξ when sprayed through the left nostril—averaged 1.81, with the minimum and maximum values being, respectively, 1.25 and 3.09; see Figure 9b. Ω values from the simulated data are plotted as the blue line in Figure 9b. The values correspond to θ=49° in the simulations, which corresponds to the measured plume angle for the spray device used in the experiments. The simulations and experiments agree when d is approximately within $\left(\left.28,50\right)\right.\;\mu$ m.

Herein (i.e., Figure 9b), note that the gray horizontal band (and the gray/green dashed bars) present data from physical experiments conducted with a realistic droplet size distribution in each actuation and do *not* have any correlation to the d values (in blue) along the horizontal axis in Figure 9b. In contrast, the Ω values along the simulation-derived blue plot are functions of d (since the simulations were monodisperse). Therefore, it is reassuring that the simulations and experiments align when d≳25 μ m, as the experimental spray shots (by design) are predominantly composed of particles within that size spectrum (see Table 1).

#### 3.3.1 Statistical evaluation of the computation-experiment comparison

Comparability between the experimental and simulated nasopharyngeal deposition trends has been evaluated by coverage analysis of the experimentally observed deposition outcome interval ( Ω∈[1.25,3.09] ; see Figure 9a—the variability in the experimental measurements is deemed attributable to spray actuation differences owing to human factors in multiple runs and device re-usage; see Section 2.3. Implicitly, 10/10 replicate experimental runs with the polydisperse LuerVax^TM^ spray ( ${\text{Dv}}_{10}$ = 20 μ m, ${\text{Dv}}_{50}$ = 43 μ m; see Table 1) fell inside the Ω interval; the exact (Clopper–Pearson; see Puza and O’Neill (2006)) 95% confidence interval (CI) for this proportion is 69.2%−100% (binomial exact for k=10 , n=10 ; with k being the number of experimental replicates that were “successes” and n being the total number of independent observations or trials). In the computational sweep of conically injected monodisperse particles for diameters exceeding 20 μ m (which per Table 1 is LuerVax^TM^’s ${\text{Dv}}_{10}$ , marking the volume-based threshold droplet diameter above which 90% of the total sprayed liquid volume is contained); i.e., with d∈[21,50] μ m (implying n = 30 sizes), 23/30 sizes (76.7%) produced deposition outcomes within Ω∈[1.25,3.09] ; the exact 95% Clopper–Pearson CI for 23/30 is 57.7%−90.1% . The coverage proportions are based on the simulated particle sizes 28−50 μ m producing deposition outcomes within the experimental Ω limits. The absolute difference in coverage between experiment and the simulation subset is, therefore, 23.3 percentage points, indicating a moderate level of similarity. The similarity, however, approaches 100% if we constrain the particle diameter space to the simulation-guided optimal ones (that lead to maximal ξ in the test domains; see Figure 8), i.e. d∈[25,45] μ m.

## 4 Discussion

### 4.1 Perspectives on the modeling approach: current limitations and future directions

The parametric recommendations (namely, optimal sprayed particle sizes 25−45 μ m and spray plume angles ≲30° ) provide practical translational insights with actionable design targets for formulation scientists and device engineers—guiding droplet size distribution tuning, formulation material, and plume-control features to maximize nasopharyngeal delivery in next-generation commercial nasal sprays. But be as it may, this study can be, however, critiqued for several limitations that relate back to the true biological realism and statistical robustness of the investigative framework—thereby conceptualizing several future study directions, as described next.

- On the structural rigidity of the anatomical domains: An important limitation herein is the assumption of structural rigidity in the nasal anatomical reconstructions. Although the geometries were built with high fidelity from medical-grade imaging, they do not factor in the temporally dynamic, elastic properties of nasal tissues, which can influence local airflow patterns and particle deposition under physiological conditions. Nasal soft-tissue compliance and transient deformations (e.g., owing to breathing cycle, muscle tone, or positional changes) can alter local airway cross-sections and consequently the near-wall velocities through geometry-flow coupling. Prior fluid–structure interaction and deformable-wall nasal studies report changes in local airflow velocities and wall shear on the order of tens of percent under physiologic wall motion or pressure loading (e.g., see Pirnar et al. (2015)), which would shift streamline patterns and the fate of borderline-inertia particles (Stk≈0.5−2.0) that govern the 25−45 μ m size band identified here for maximal ξ . Consequently, the location and magnitude of peak ξ (especially near geometric bottlenecks) could change when compliance is included. It is therefore reasonable to frame our results as mechanistic, geometry-specific predictions; a planned extension could be to perform sensitivity runs with representative compliant wall models or parametrically perturbed geometries to see how intra-airway regional deposition maps (exemplified in Figure 8) might move under physiologically plausible deformations.
- On the effects of mucociliary clearance: Our simulations capture the intra-airway spatial transport and wall impaction of sprayed particles over a time-window of 0.35 s (see Section 2.2) and do not account for mucociliary clearance, which operates on much longer time scales (namely, minutes to hours; e.g., see Shang et al. (2019)). From a physiological perspective, the mucociliary transport (comprising creeping Stokes flow-like dynamics along the airway walls) is expected to progressively remove deposited material from the nasopharynx, reducing regionally retained mass and therapeutic residence time. Prior reported mucociliary clearance rates correspond to downstream surface transit speeds of ≈5−20 mm/min (Fahy and Dickey, 2010), which can often substantially lower effective dose available for local action over clinical time-scales. By not incorporating this effect in the current modeling framework, our reported ξ values exclusively represent initial targeted deposition (i.e., particle counts delivered to tissue) rather than retained dose over time. Future work should hence couple direct deposition outputs with clearance models (e.g., by tracking advective transport with mucociliary stream; see Knowles and Boucher (2002); Shang et al. (2019)) or time-resolved mucociliary surface transport simulations to estimate clinically relevant retained mass as a function of dosing frequency.
- On the monodisperse nature of the simulated sprays: Another key nuance is the absence of particle size distribution consideration in the simulations. For computational control, we used monodisperse particle injections to map size-resolved behavior. Actual spray products offer a heterogeneous size distributions (polydispersity) of aerosols and microdroplets. Polydispersity alters volume-weighted deposition because larger droplets contribute disproportionately to mass deposition while smaller fractions may bypass the nose (Finlay, 2001). However, this study was designed to systematically identify which specific particle sizes are most suited at *directly* reaching the nasopharynx through the spraying action; (it is expected that) the information could then guide the design of real sprays with their particle sizes geared toward the precise findings of this study.
- On toxicological suitability: Next, we have overlooked (for now) the potential chemical and/or biological interactions within the nasal mucosa, such as mucociliary clearance or enzymatic activity, which can impact deposition (and therapeutic) efficacy over time. Another somewhat-related and crucial consideration involves the toxicological safety associated with increased targeted deposition. While larger particles like the ones between 25–45 μ m are demonstrably likely to directly deposit at the nasopharynx (through spraying) and thus effectively deliver the therapeutic agents, the size of the particles and the material density of the formulation could have implications for safety profiles. Particles within specific size ranges may present risks of localized irritation or trigger immune responses, and the formulation properties may necessitate comprehensive toxicological assessment to avert adverse effects, including inflammation or unintended tissue damage. For the latter, it should however be noted that all other parameters remaining same, the 1 g/mL water-like formulation constitution did give the highest mean nasopharyngeal deposition.
- On the plume angle in the experimental test case: A specific limitation of the experimental validation is that θ=49° for LuerVax^TM^, and the compared simulated data, therefore, was for the same plume angle (note Figure 9’s zoom-out from panels a to b). The validated data is hence for a θ outside of the optimal domain θ≲30° . While the validation lends support to the reliability of the simulated physics, future studies should test other spray products ideally in conformity with the prescribed optimality ranges for {d,θ,ρ} , to fully establish the robustness of the numerical findings.
- On the generalization constraints in the experimental validation: The experimental validation exercise was intentionally limited to a single 3D-printed anatomical cast (namely, of ${\text{AG}}_{2}$ ) and one nasal spray device (LuerVax^TM^ from Aptar Pharma), in view of practical constraints related to optical imaging requirements and possible human actuation errors from repeat runs. ${\text{AG}}_{2}$ was selected as it clearly exhibited the higher nasopharyngeal deposition (ξ) in our simulations (comparing row-wise across panels in Figure 6; check the color scales) and consequently was expected to serve as a conservative, high-signal test case for validating the flow–particle physics and right/left nostril deposition asymmetry. It should be emphasized that the experimental campaign was designed to representatively validate the computation-derived mechanistic trends—not to demonstrate population-level generalizability. The narrow experimental scope may arguably constrain extrapolation to broader device variability; this should motivate future work to expand validation across additional spray products and subject anatomies.
- On contextualizing the reported maximal ξ : The globally averaged nasopharyngeal deposition efficiency map (Figure 8) suggests a maximal mean ξ≈11.4% if d∈[25,45] μ m and θ≲30° (marked by the solid red rectangle in Figure 8a); this lies at the upper end of typical posterior-nasal deposition fractions reported in the literature (Laube et al., 2024; Basu et al., 2020). Numerous *in vitro* and *in vivo* gamma scintigraphy and surrogate-model studies report nasopharyngeal or posterior nasal deposition in the low single digits to low tens of percent depending on device, spray technique, droplet size distribution, and anatomy (typical ranges being 1−15 %; e.g., see Djupesland (2013)). Well-directed sprays with optimized nozzle placement have previously produced posterior deposition values in the 5−12 % range in controlled studies (Basu et al., 2020), which is comparable to the peak values we predict here for the optimized parametric window. As a targeted-design performance benchmark, the simulated 11.4% thus represents a physiologically plausible and pharmaceutically meaningful improvement relative to generic, non-optimized administration (which commonly yields lower single-digit posterior deposition), provided device and formulation follow the identified design guidelines. We emphasize, however, that (i) reported deposition fractions can vary with subject anatomy, (ii) our value reflects initial deposition (not retained dose over time; see the prior discussion on mucociliary clearance effects), and (iii) the finding corresponds to a limited range of particle sizes with equal weightage given to each in the monodisperse simulation protocol (to pinpoint the most optimal particle sizes for targeted nasopharyngeal deposition), unlike heterogeneous polydisperse sprays.
- On the test cohort size: Finally, the study uses a restricted cohort of two test subjects with four representative anatomical airspace pathways. It clearly does not capture a statistically significant range of inter-individual variability and inhalation patterns (beyond the simplified relaxed inhalation scenario); consequently, the generalizability of the current findings across wider populations is yet to be established.

### 4.2 The main takeaways

Backed by experimentally validated computational fluid dynamics simulations, this investigation emphasizes the critical role of optimizing spray device and formulation parameters to enhance targeted drug delivery within the complex anatomical landscape of the human nasal cavity. Key findings include:

- Optimal particle sizes: Higher formulation densities increase particle inertial effects, shifting deposition loci toward anterior regions of the nasal airspace, owing to inertial impaction. When averaged across all formulation densities and airway-specific deposition trends, the particles within the size range of approximately 25–45 μ m, combined with optimized spray angles (note the next bullet point), significantly maximize deposition at the nasopharynx (a key initial infection tissue site); see Figures 8a,b. Note also Table 1 for the droplet size distributions in selected state-of-art spray products. The ${\text{Dv}}_{50}$ (a spray characteristic representing the volume median diameter of droplets in a spray plume (Finlay, 2001)) for BiVax 200^TM^ and LuerVax^TM^ (both Aptar Pharma products; see Table 1) align well with our model predictions for optimal particle size range.
- Plume angle optimization: Narrower spray plume angles (θ) are more effective in concentrating delivery toward the nasopharynx, reducing off-target deposition and improving therapeutic precision; see the global averaged map in Figures 9a,b. For example, with d∈[25,45]μ m, if θ≤ 45 ${\;}^{{}^{\circ}}$ —then that gives a 45% higher mean ξ than its global mean (see Figures 8a,b). Such a θ (at its extremal value) would also render the conceptualized product tantalizingly close to LuerVax^TM^ in terms of specification (see Table 1). A more ambitious modification with θ≤ 30 ${\;}^{{}^{\circ}}$ for the same d span improves ξ by 76% compared to its global mean in the comprehensive parametric space explored.
- Parameter synergy: As a specific prescription, the combination of particle sizes between 25–45 μ m and spray plume angles ≤ 30 ${\;}^{{}^{\circ}}$ yields the highest average deposition efficiencies ( ∼ 11.4%).

In conclusion, this *in silico* physiology-guided computational study provides a rational, simulation-informed design recommendations for spray-based intranasal drug delivery systems—to achieve maximal targeted deposition of pharmaceutics at the nasopharynx (a key infection launch site for several respiratory pathogens). The parametric findings are, however, grounded in simulation data from only two representative subjects, with sprayed transport of particles analyzed within four nasal pathways. Future work should focus on: enhancing the test cohort size, incorporating tissue compliance effects, expanding to diverse anatomical variants, and conducting comprehensive toxicological assessments and safety checks for the optimized formulations and devices; the latter is especially critical in view of the elevated tissue deposition expected from the augmented spray designs.

## Data Availability

All essential information is contained in the article. Supplementary Material (including anatomical geometries, simulation files, postprocessing spreadsheets, and programming codes) are available via the open access repository figshare, with doi: 10.6084/m9.figshare.29640497. The reader may also contact the corresponding author for any relevant data.
