# Supplementary material for: Mechanics-guided parametric modeling of intranasal spray devices and formulations for targeted drug delivery to the nasopharynx
Source: Front Drug Deliv. 2025 Dec 12;5:1721960. doi: 10.3389/fddev.2025.1721960 (PMC12741747; doi:10.3389/fddev.2025.1721960)
Supplement: Supplementary file 1 [file DataSheet1.pdf]

Supplementary Material

1 SUPPLEMENTARY DATA

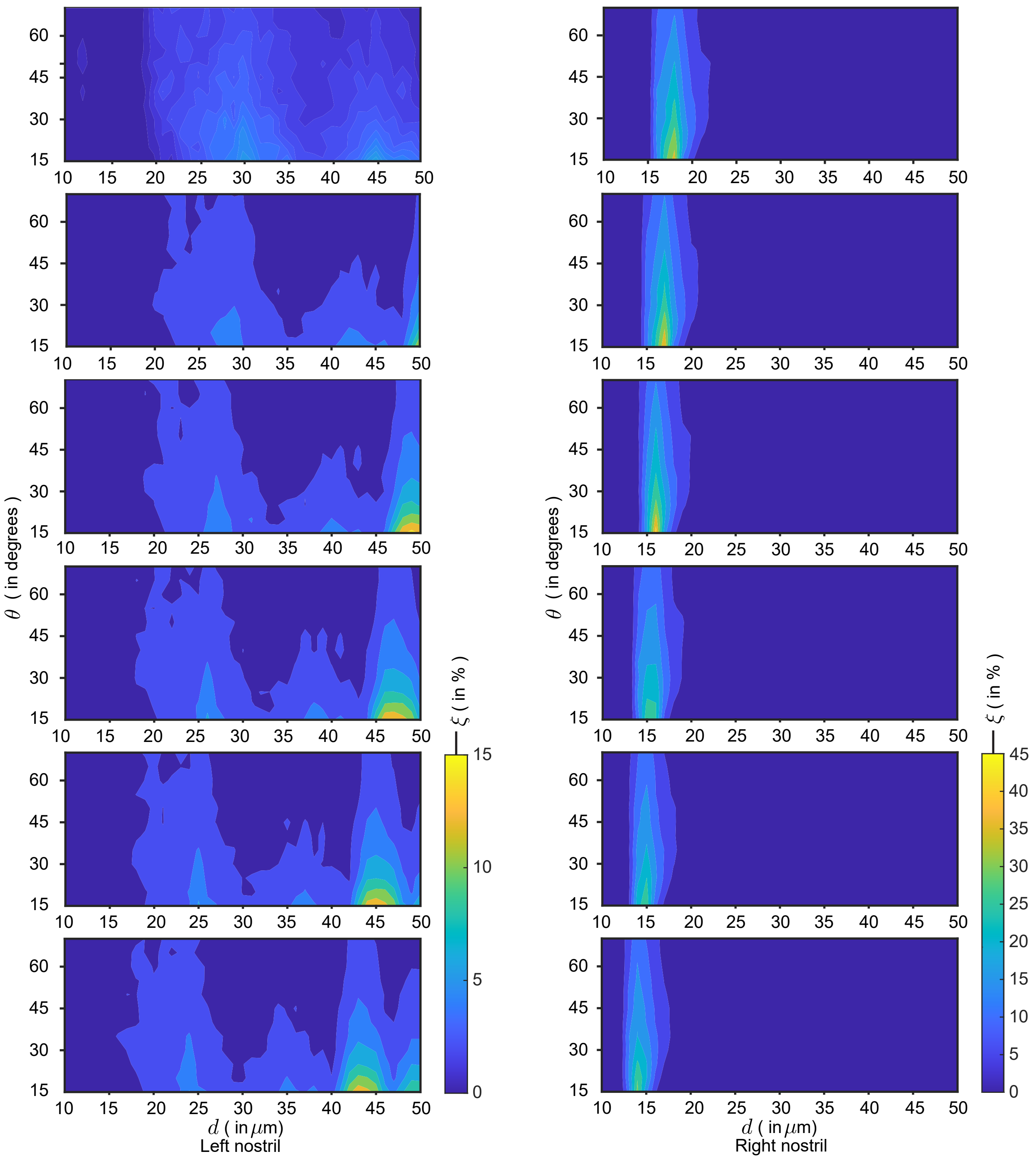

Figure S1. Simulated nasopharyngeal deposition trend for AG_1: Contour plots for nasopharyngeal deposition rate (as in, what fraction of the monodisperse particles end up depositing at the nasopharynx; represented as ξ) as a function of the spray plume angles (θ, recorded along the horizontal axis) and particle diameters (d, recorded along the vertical axis). Owing to the effect of inertial impaction on downwind penetration, the optimal parametric region (for maximal ξ) gradually shifts toward the left side of the contour map with increasing formulation density ρ. Top-to-bottom: $\rho \in [1.0, 1.5]$ g/ml, with an increment of 0.1 g/ml in successive rows. Left column: left nostril data; right column: right nostril data.

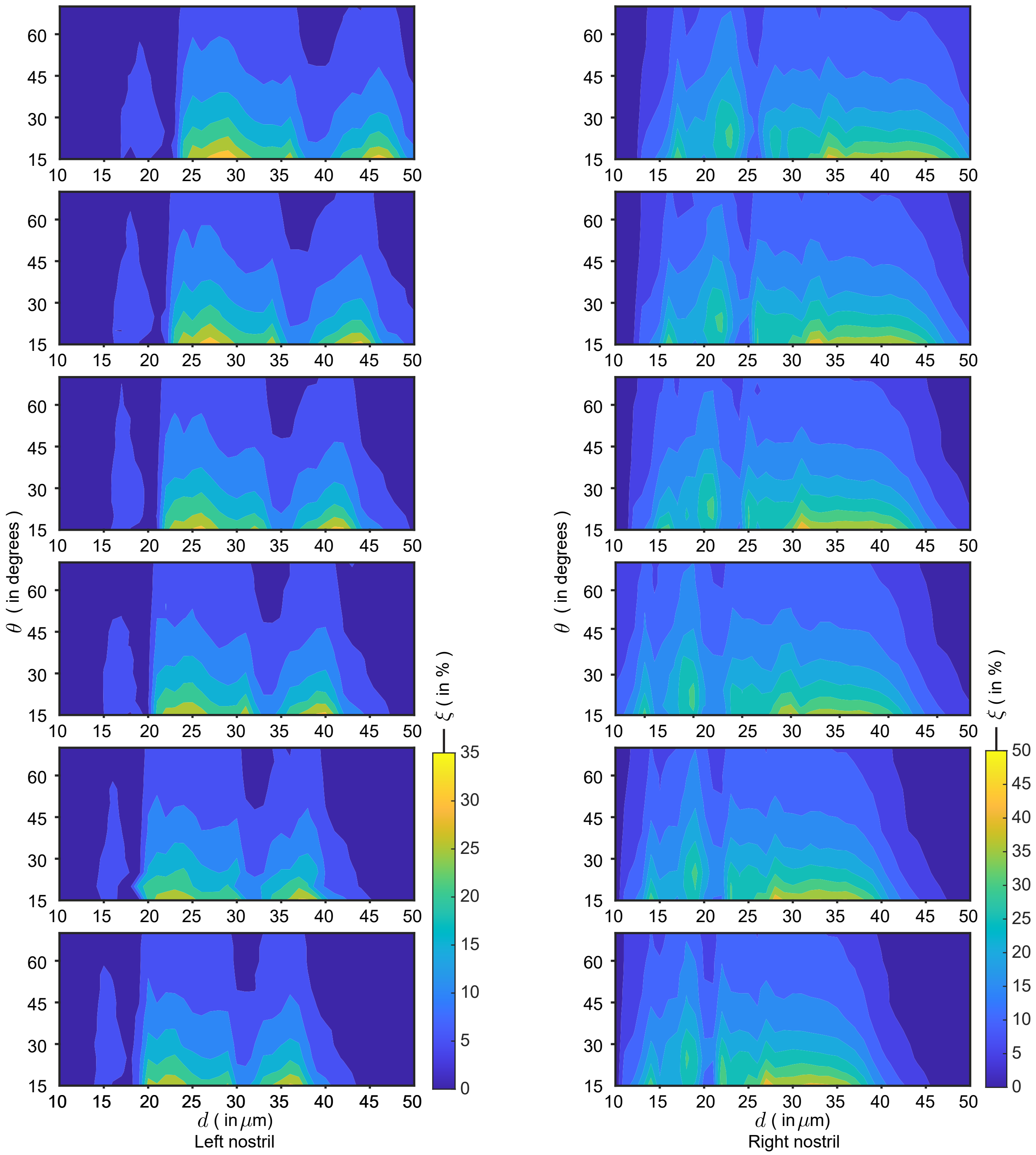

Figure S2. Simulated nasopharyngeal deposition trend for AG_2: Contour plots for nasopharyngeal deposition rate (as in, what fraction of the monodisperse particles end up depositing at the nasopharynx; represented as ξ) as a function of the spray plume angles (θ, recorded along the horizontal axis) and particle diameters (d, recorded along the vertical axis). Owing to the effect of inertial impaction on downwind penetration, the optimal parametric region (for maximal ξ) gradually shifts toward the left side of the contour map with increasing formulation density ρ. Top-to-bottom: $\rho \in [1.0, 1.5]$ g/ml, with an increment of 0.1 g/ml in successive rows. Left column: left nostril data; right column: right nostril data.
